# Polymer-Clay Nanocomposites for the Uptake of Hazardous Anions

**DOI:** 10.3390/nano14050467

**Published:** 2024-03-04

**Authors:** Huaibin Zhang, Wenyan Huang, Sridhar Komarneni

**Affiliations:** 1 Department of Ecosystem Science and Management and Materials Research Institute, 204 Energy and the Environment Laboratory, The Pennsylvania State University, University Park, PA 16802, USA; zhanghb110@gmail.com (H.Z.); ellahwy@cczu.edu.cn (W.H.); 2 Jiangsu Key Laboratory of Environmentally Friendly Polymeric Materials, School of Materials Science and Engineering, Changzhou University, Changzhou 213164, China

**Keywords:** nanocomposites of montmorillonite, anion exchange, kinetics, anion selectivity, polydiallyldimethylammonim cations, polymer

## Abstract

Polymer intercalated clay nanocomposites were prepared from various montmorillonites (Mt) and a polymer, polydiallyldimethylammonim (PDDA) chloride. X-ray diffraction (XRD) analysis of the above polymer intercalated nanocomposites showed either no crystalline peaks or very broad peaks with the intercalation of PDDA polymer in the interlayers, probably as a result of exfoliation of the clay layers. Infrared spectroscopy revealed the presence of PDDA in all the clay nanocomposite materials. The maximum adsorption capacities of nitrate, perchlorate, and chromate by one of the polymer intercalated nanocomposite materials prepared from montmorillonite, Kunipea were 0.40 $mmol\cdot g^{-1}$, 0.44 $mmol\cdot g^{-1}$ and 0.299 $mmol\cdot g^{-1}$, respectively. The other two polymer intercalated nanocomposites prepared with montmorillonites from Wyoming and China showed very good adsorption capacities for perchlorate but somewhat lower uptake capacities for chromate and nitrate compared to the nanocomposite prepared from montmorillonite from Kunipea. The uptake of nitrate, perchlorate and chromate by the polymer intercalated nanocomposites could be well described using the Freundlich isotherm while their uptake kinetics fitted well to the pseudo-second-order model. The uptake kinetics of nitrate, perchlorate, and chromate were found to be fast as equilibrium was reached within 4 h. Moreover, the uptakes of chromate by polymer intercalated nanocomposites were found to be highly selective in the presence of ${Cl}^{-}$, ${SO}_{4}^{2-}$ and ${CO}_{3}^{2-}$, the most abundant naturally occurring anions.

## 1. Introduction

Industrial, mining, refinery, and chemical storage sites pose risks of releasing harmful pollutants to the environment. Of the many toxic organic and inorganic substances that can be generated at such sites, anionic pollutants are highly mobile in soils and can be leached into groundwater, causing environmental and health damage. Among all these anions, chromate is highly toxic and is a worldwide problem. A slight increase in chromate concentration causes environmental and health problems due to its high toxicity [1,2], mutagenicity [3] and carcinogenicity [4].

Nitrate is an essential nutrient for plants and plays a key role in agricultural crop production, which is very important for the sustainability of agriculture [5]. Animal excreta is also one of the most common sources of nitrate [6]. However, the contamination of groundwater with nitrates can result from the excessive use of nitrate fertilisers and from leaky sewage treatment systems. In addition, nitrate can cause eutrophication and infectious diseases, such as cyanosis and cancer in the alimentary canal [7]. Perchlorate has been widely used in rocket fuels, propellants, explosives and some consumer products [8,9]. Ingestion of high doses of perchlorate impairs the development of the skeletal system; the central nervous system of infants is affected, and the uptake of iodine into the thyroid gland may also be affected [10]. The US Environmental Protection Agency (US EPA) has set the maximum contaminant level (MCL) for chromate, nitrate and perchlorate at 0.1 ${mg\cdot L}^{-1}$, 10 ${mg\cdot L}^{-1}$ and 4 ppb, respectively in drinking water [11]. Therefore, there is a clear need for the development of cost-effective selective anion exchangers to remove chromate, nitrate, and perchlorate from water.

Many materials and technologies have been developed and tested for the removal of chromate, nitrate, and perchlorate. These include electrochemical treatment [12,13,14,15], membrane filtration [16,17,18,19], biodegradation [20,21], activated carbon [22,23,24], ion exchange resin [25,26,27,28,29], zeolite [30,31,32,33], organoclays [34,35,36] etc. A previously published paper by Stefan Dultz et al. [36] reports 0.37 and 0.26 mol·kg^−1^ chromate removal by vermiculite modified with Hexadecylpyridinium (HDPy) and Hexadecyltrimethylammonium (HDTMA), respectively. Fethiye Gode et al. [37] also prepared Poly(acrylic acid)–Bentonite Composite, which shows that the maximum capacity of chromium removal was 29.55 mg·$g^{-1}$ at 25 °C, which was much higher than that of the vermiculite (1.3 mg·$g^{-1}$). Moreover, Octadecyltrimethylammonium (ODTMA) intercalated vermiculite was synthesised by Suramya et al. [38] and reported a 79.45–91.02% removal rate of chromate using the initial concentration range of 2 g·$L^{-1}$ to 4 g·$L^{-1}$.

In a previous study, a synthetic fluoromica was intercalated with polydiallyldimethylammonim (PDDA), and they suggested its potential for anion uptake [39]. Three cationic polyelectrolytes, polyethylenimine (PEI), poly(allylamine hydrochloride) (PAH), and PDDA, were intercalated into sodium fluortetrasilisic mica (Na-TSM). They found that PDDA, which had the lower positive charge density, adopted a coiled conformation within the interlayers of the clay and found that this composite had an anion-accepting ability. An anionic dye was used for testing anion exchange, which was found to be intercalated into the hydrated galleries in which the PDDA strands were coiled [39].

However, most of these studies did not examine PDDA-intercalated montmorillonite (PDDA-Mt) for systematic exchange uptake of chromate. Moreover, the adsorption behaviour of PDDA-Mt for different anions and the mechanism were not clear in the previous studies. Therefore, in this study, we have investigated the exchange, selectivity, kinetics and mechanism of chromate, nitrate, and perchlorate by PDDA-Mt nanocomposite. The novelty of this study is that we not only determined selective anion uptake by PDDA-Mt nanocomposite but also proposed that the mechanism of anion uptake is due to the presence of neutral polymer with ${Cl}^{-}$, which is entrapped by negatively charged clay layers and polymer cations in the interlayers and these anions participated in the exchange.

## 2. Experimental

### 2.1. Materials

The Na-montmorillonite (Na-Mt) (Kunipia-F) used in this study was obtained from Kunimine Industries Co., Ltd., Tokyo, Japan and has a cation exchange capacity (CEC) of 1.05 $mmol\cdot g^{-1}$. This montmorillonite will be referred to as Mt-J hereafter. Another montmorillonite (Bt-3) was obtained from the Zhejiang Changan Renheng Technology Co., Ltd., Huzhou, Zhejiang, China, and it has a cation exchange capacity (CEC) of 0.85 $mmol\cdot g^{-1}$. This sample will be referred to as (Mt-C) below. Another montmorillonite from Wyoming was obtained from the Clay Minerals Society, and this montmorillonite is referred to as (Mt-W). Wyoming montmorillonite was used because it is the most abundant montmorillonite from Wyoming, USA and has a CEC of 0.85 $mmol\cdot g^{-1}$. The PDDA (Polydiallyldimethylammonim chloride) (Mw < 100,000) was purchased from Sigma-Aldrich as a 35.0 wt% aqueous solution. Water used in all the experiments was deionised using a Nanopure water treatment system.

### 2.2. Preparation of PDDA Intercalated Montmorillonites (Mt)

**Figure 1:**
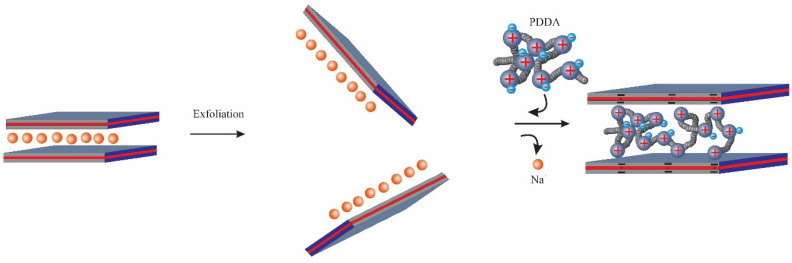
Schematic synthesis of the clay-PPDA nanocomposite.

For the Synthesis of PDDA-Mt nanocomposite, clay was first exfoliated or delaminated by stirring in water as follows: 0.75 g of Mt was added into 74.25 g of deionised water and the suspension was stirred for 24 h at room temperature to ensure that the Mt was sufficiently delaminated. Montmorillonite was gradually added to deionised water by adding small amounts of Mt with stirring. An aqueous solution of PDDA, with a total molar amount equal to 2 times the cations exchange capacity (CEC) of montmorillonite, was then added to the above suspension. After the equilibrium, the suspension was centrifuged, and the resulting product was washed twice with 100 g of water to remove excess polymer [39]. Finally, the sample was dried at 333 K for 1 to 2 days and ground to a powder. The PDDA nanocomposites obtained in this way with Mt from Japan, Mt from China and Mt from Wyoming are referred to as P-Mt-J, P-Mt-C and P-Mt-W, respectively (Figure 1.)

### 2.3. Sample Characterization

#### 2.3.1. Powder X-ray Diffraction

XRD patterns were recorded with a Scintag X-ray diffraction unit operated at a voltage of 35 kV and 30 mA using Cu Kα radiation in the 2θ range of 5–10 at a scan speed of 2° min^−1^. The crystalline phases and the nature of their crystallinity were determined by XRD measurements on all montmorillonites and the PDDA-Mt nanocomposites.

#### 2.3.2. Infrared Spectroscopy

Fourier transform infrared (FTIR) spectra were acquired using the KBr pressed disk technique on a Thermo Nicolet 6700 FTIR spectrophotometer. The FTIR spectra in the range of 4000–400 ${cm}^{-1}$ were recorded with a resolution of 4 ${cm}^{-1}$. Measurements were performed at room temperature.

### 2.4. Anion Uptake Studies

The uptake of perchlorate using the as-prepared PDDA-Mt nanocomposites thus prepared was performed as follows: 25 mL of 1 mM sodium nitrate (Alfa Aesar chemical), sodium perchlorate (Alfa aesar chemical) or sodium chromate (Alfa Aesar chemical) solution was added to 50 mg of the nanocomposite material in centrifuge tubes to perform a batch equilibration study. After mixing the tubes by hand for one or two minutes, they were mixed on a shaker for 24 h. After 24 h equilibration, the solid and dissolved phases were separated by centrifugation. Then, 15 mL of each solution was collected in clean vials for anion analysis. All anion uptake experiments were conducted using triplicates. Anion concentrations were monitored using the Dionex DX-500 ion chromatograph equipped with an AS40 autosampler, a 4-mm AS22 column, a 4-mm ASRS 300 suppressor, and a DS4 detection stabiliser. The stabiliser used a current of 40 mA and a temperature of 25 °C. The eluent concentration used was 4.8 M Na_2_CO_3_ and 1.0 M Na_2_CO_3_ and a 1000 μL sample loop was used for these anions analyses. The amount of adsorbed perchlorate qt ($mg\cdot g^{-1}$) at any time was calculated as follows:

(1) $$q_{t}=\frac{V(C_{0}-C_{t})}{m}$$

where $C_{0}$ is the initial concentration $\left(g\cdot {mL}^{-1}\right)$ of perchlorate, $C_{t}$ is concentration $\left(g\cdot {mL}^{-1}\right)$ of perchlorate at any time, V is the perchlorate solution volume $\left(mL\right)$ and m is the mass of adsorbent $\left(g\right)$ [40,41].

### 2.5. Kinetics Studies

The kinetics of the anion uptake by PDDA-Mt nanocomposites was determined as follows: 50 mg of each sample was mixed with 25 mL of anion solution at a concentration of 1 mM in centrifuge tubes. The dispersions were shaken for 5 min, 30 min, 2 h, 4 h, 8 h, and 24 h on a shaker. The kinetics experiments were conducted in triplicate. The tubes were centrifuged to separate the solid and solution phases. Then, 15 mL of each solution was collected in clean vials for anion analysis. After equilibration, the suspensions were centrifuged to separate the solid from the liquid phase, and the liquids were collected to determine the concentration of nitrate and perchlorate using Dionex DX-120 ion chromatograph as described above [40,41].

### 2.6. Isotherm Studies

Perchlorate, chromate, and nitrate anion exchange isotherms were determined after equilibration of 50 mg Clay-PDDA nanocomposite with 25 mL of 0.1, 0.3, 0.5, 0.8, and 1.0 mM anion solution by shaking for 24 h. All the anion exchange-isotherm experiments were also conducted in triplicate. The tubes were centrifuged to separate solid and solution phases, and then 15 mL of each solution were collected in clean vials for perchlorate, chromate and nitrate analysis as above [40,41].

### 2.7. Effect of Competitive Anions and Determination of $K_{d}$ Values

The effect of the competing anions such as ${Cl}^{-}$, ${SO}_{4}^{2-}$ or ${CO}_{3}^{2-}$ was investigated by equilibrating 0.05 g each of PDDA-Mt nanocomposite material by mixing with 25 mL of 1 mM of each anion with 10 mM of NaCl or 5 mM of ${Na}_{2}{SO}_{4}$ or 5 mM of ${Na}_{2}{CO}_{3}$ in centrifuge tubes. The suspensions were shaken for 24 h on a shaker. The tubes were then centrifuged, and 15 mL of each solution was collected in clean vials for analysis. The distribution coefficient $\left(K_{d}\right)$ is defined as the ratio of the number of sorbed ions per gram of solid to the number of ions remaining per millilitre of solution and is expressed as millilitres per gram. $K_{d}$ value and removal efficiency (anion removal, %) were calculated as follows:

(2) $$K_{d}=\frac{C_{0}-C_{e}}{C_{e}}\times \frac{V}{m}$$

(3) $$anion\;removal,\;\%={100(C}_{0}-C_{e})/C_{e}$$

where $C_{0}$ and $C_{e}$ are the ion concentrations in the initial solution and in the solution after equilibration with nitrate, perchlorate, or chromate anion, V is the volume of solution in mL and m is the mass of sorbent $\left(g\right)$ [40,41].

## 3. Results and Discussion

### 3.1. X-ray Diffraction

**Figure 2:**
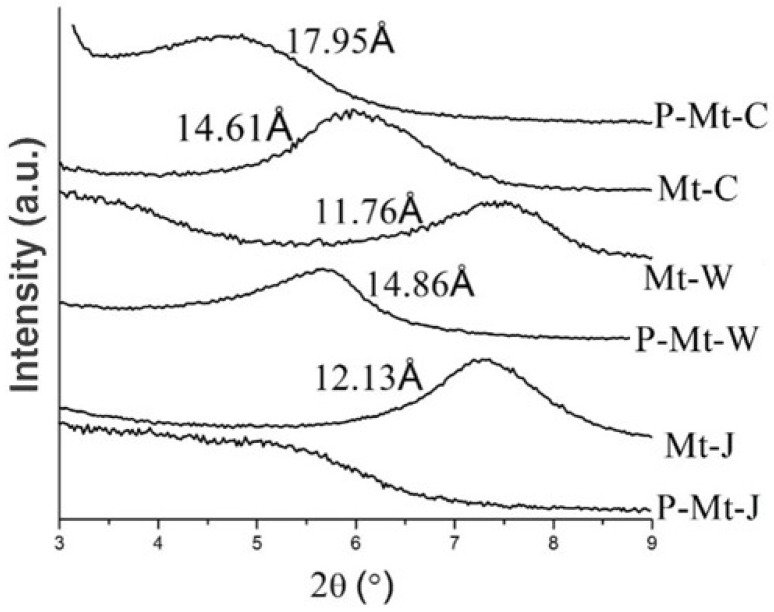
X-ray diffraction patterns of Wyoming montmorillonite (Mt-W) and the PDDA intercalated Mt-W (P-Mt-W), montmorillonite from China (Mt-C) and the PDDA intercalated Mt-C (P-Mt-C) and montmorillonite from Kunipea, Japan (Mt-J) and the PDDA intercalated Mt-J (P-Mt-J).

Figure 2 shows the X-ray diffraction patterns of montmorillonites from Wyoming (Mt-W), China (Mt-C) and Japan (Mt-J) before and after treatment with PDDA. Mt-W, Mt-C and Mt-J showed broad peaks at d value = 14.81 Å, 14.61 Å and 12.13 Å, respectively, corresponding to the main crystal plane (001) of montmorillonite. After intercalation with PDDA, the $d_{001}$ basal spacings of Mt-W and Mt-C shifted to higher values with broader peaks, which were 14.86 Å and 17.95 Å, respectively (Figure 2). These larger $d_{001}$ values indicate that the interlayer space was expanded to 14.86 Å as the PDDA intercalated into the montmorillonite’s interlayers of Mt-W. In Mt-J, the increased width of the peak suggests that the size of the crystalline domains along the stacking axis decreased after intercalation. Also, the asymmetric shape of the peak suggests that there are multiple phases with different d-spacings in these composites.

### 3.2. Infrared Spectroscopy

**Figure 3:**
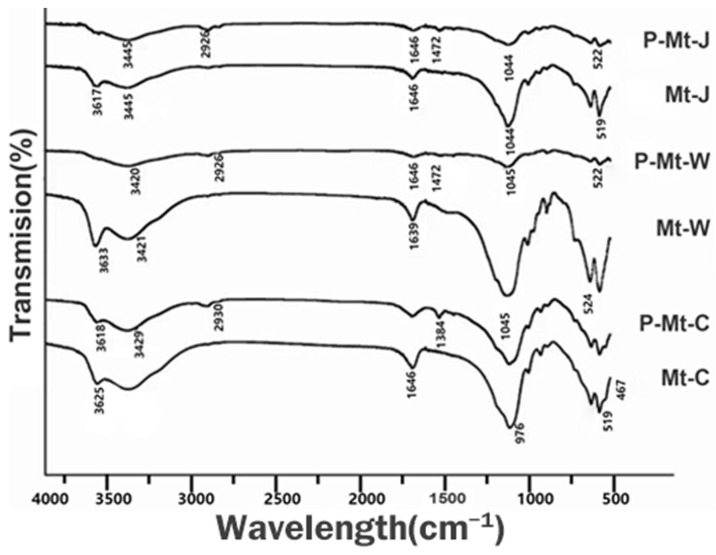
Infrared spectra of Mt-J, the intercalated Mt-J with PDDA (P-Mt-J), Mt-C, the intercalated Mt-C with PDDA (P-Mt-C), Mt-W and the intercalated Mt-W with PDDA (P-Mt-W).

Infrared (IR) spectroscopy was used to detect intercalation of PDDA in different montmorillonites. The IR spectra of different montmorillonites before and after treatment with PDDA are shown in Figure 3. Infrared spectra of the three montmorillonites show the high-intensity absorption bands at 1100 to 900 ${cm}^{-1}$ (Si-O in-plane stretching) and 529 ${cm}^{-1}$ (Si-O bending vibrations) and broad bands at 3600 to 3700 ${cm}^{-1}$ and 1639 ${cm}^{-1}$, which is attributed to the stretching and bending vibrations for the hydroxyl groups of water molecules present in the montmorillonite [42]. The difference between the FTIR spectra of montmorillonites before and after PDDA intercalation (Figure 3) is the appearance of new peaks at 1472 ${cm}^{-1}$ (C=C and C=N in-plane vibration) 1384 ${cm}^{-1}$ (C-H deformation vibration in ring) [43] and 2930 ${cm}^{-1}$ (C-H stretching vibration) [44]. Thus, the FTIR spectra prove that C=C and C=N bonds representing PDDA are present in the montmorillonites after PDDA intercalation.

### 3.3. Perchlorate, Chromate, and Nitrate Uptake by Mt-PDDA Nanocomposites

Perchlorate, chromate, and nitrate uptakes by the Mt-PDDA nanocomposites are shown in Table 1. From the results presented in Table 1, it is obvious that the highest uptakes of perchlorate, chromate and nitrate were by P-Mt-J with 0.44 $mmol\cdot g^{-1}$, 0.42 $mmol\cdot g^{-1}$ and 0.30 $mmol\cdot g^{-1}$, respectively. P-Mt-C showed uptakes of 0.4 $mmol\cdot g^{-1}$, 0.41 $mmol\cdot g^{-1}$ and 0.24 $mmol\cdot g^{-1}$ of perchlorate, chromate, and nitrate, respectively. In the case of the P-Mt-W, the uptakes of perchlorate, chromate and nitrate were 0.35 $mmol\cdot g^{-1}$, 0.34 $mmol\cdot g^{-1}$, 0.19 $mmol\cdot g^{-1}$, respectively. Thus, the above results show that the lowest uptakes of perchlorate, chromate and nitrate were by P-Mt-W, and the highest uptakes were by P-Mt-J. It appears that the Mt-PDDA nanocomposite, P-Mt-J, entrapped more PDDA than the other two montmorillonites, as the former showed higher anion uptake capacity. The entrapped amount of PDDA may be related to the cation exchange capacities (CECs) of the montmorillonites because the positively charged PDDA balances the negative charges of the montmorillonites. The CECs of the montmorillonites are in the following order: Mt-J (1.05 $meq\cdot g^{-1}$) > Mt-C (0.85 $meq\cdot g^{-1}$) > Mt-W (0.75 $meq\cdot g^{-1}$) while the anion uptake by the PDDA intercalated montmorillonites followed the same order as follows: P-Mt-J > P-Mt-C > P-Mt-W. The XRD patterns of all the Mt-PDDA nanocomposite materials (Figure 2) did not change after the uptake of nitrate or other anions.

### 3.4. Adsorption Equilibrium Isotherms

**Figure 4:**
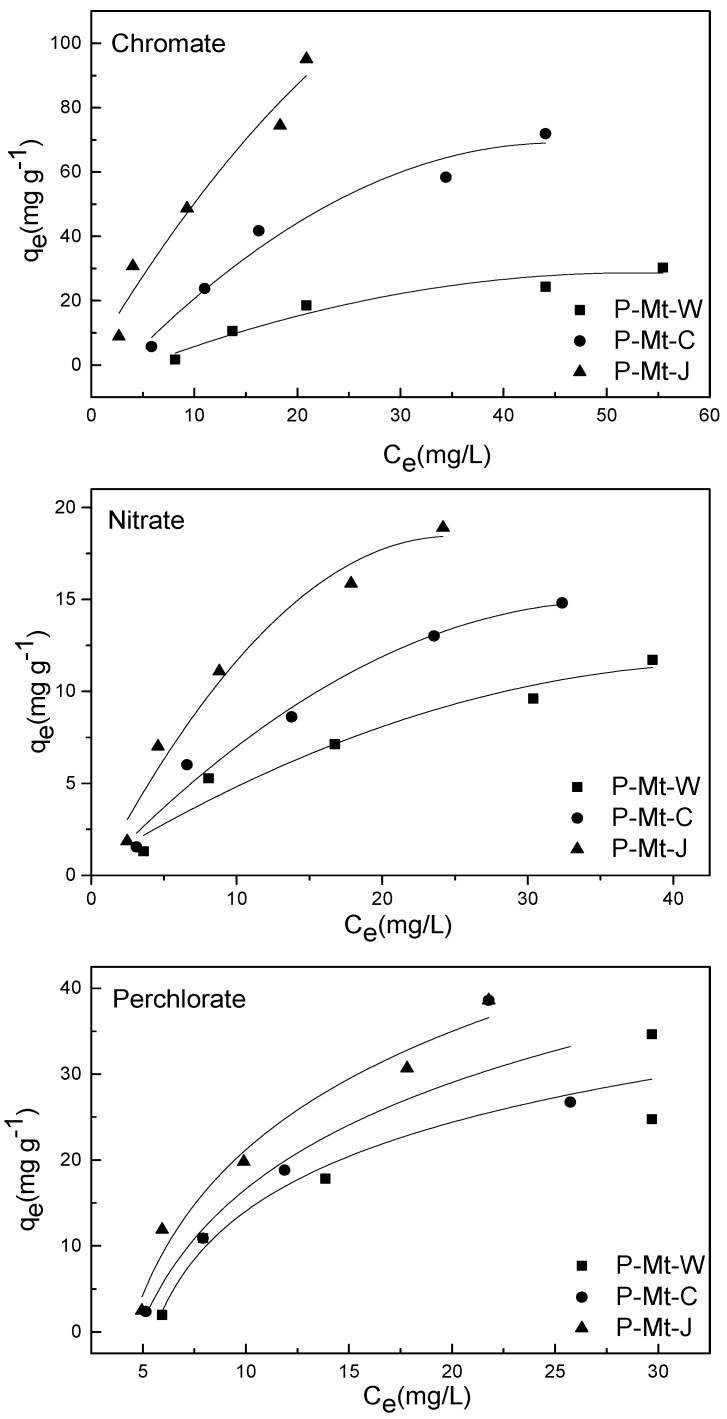
Anion exchange isotherms of P-MT-W, P-MT-C and P-Mt-J nanocomposites treated with perchlorate, chromate, or nitrate solutions of different concentrations.

The uptake of perchlorate, nitrate and chromate by P-Mt-W, P-Mt-C and P-Mt-J Mt-PDDA nanocomposites using different initial concentrations of anions is shown in Figure 4. The amount of anion exchanged by P-Mt-W, P-Mt-C or P-Mt-J nanocomposites versus the concentration of anion remaining in the equilibrium solution was plotted as isotherm in Figure 4.

Here, Langmuir and Freundlich’s isotherms were used to evaluating the adsorption behaviour for various solid–liquid systems. Based on Langmuir’s theory, there are specific adsorption sites on the surface that become saturated after monolayer adsorption of sorbate is achieved, and there is no significant interaction between adsorbed species [41,45,46]. The Langmuir model is expressed as follows:

(4) $$\frac{C_{e}}{q_{e}}=\frac{1}{b\cdot Q_{0}}+\frac{C_{e}}{Q_{0}}$$

where $q_{e}$ is the amount of perchlorate, chromate or nitrate adsorbed per unit weight of adsorbent ($mg\cdot g^{-1}$), $C_{e}$ is the concentration of anion adsorbed in the liquid phase at equilibrium ($mg\cdot g^{-1}$), b is the Langmuir constant which is related to adsorption capacity, and energy of adsorption ($L\cdot {mg}^{-1}$) and $Q_{0}$ is attained concentration corresponding to monolayer coverage [41,45,46].

The Freundlich isotherm is usually used to show non-ideal adsorption of the distribution of active centres or characteristics of heterogeneous surfaces [47,48]. This model states that reactions take place in several sorption sites, and as the amount of solute adsorbed rises, the binding surface energy decreases exponentially, which means multilayer sorption [49,50,51]. Freundlich isotherm is described as:

(5) $$\log q_{e}=\log k+\frac{1}{n}\log C_{e}$$

**Figure 5:**
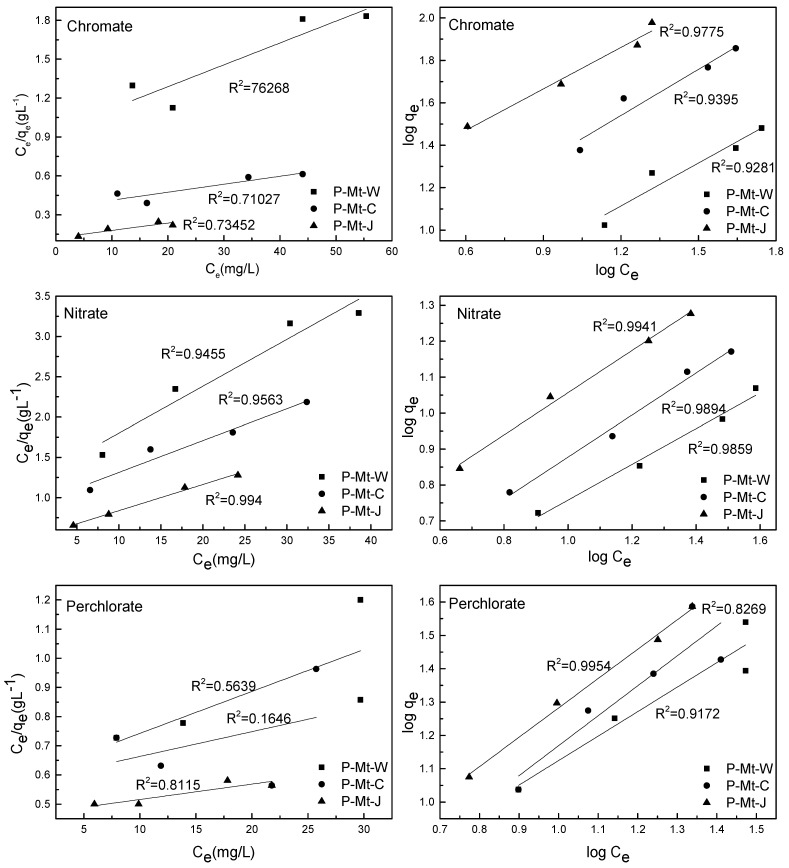
Langmuir (**left**) and Freundlich (**right**) isotherms for perchlorate, chromate and nitrate uptake by P-MT-W, P-MT-C and P-Mt-J nanocomposites.

By plotting $\log q_{e}$ versus $\log C_{e}$, the coefficients, k and n can be determined if a straight line is obtained. The Freundlich isotherms for perchlorate, chromate, and nitrate adsorption by the present montmorillonite-PDDA nanocomposites are shown in Figure 5. Correlation coefficients and isotherm constants were calculated using Langmuir and Freundlich equations and are shown in Table 2. It is evident from higher correlation coefficients that the adsorption of perchlorate, chromate and nitrate can be better described by the Freundlich isotherm than the Langmuir isotherm.

Since the Langmuir model did not fit well with the adsorption process, it is not a monolayer adsorption, but it may be a multilayer adsorption. The reason may be that it is not a uniform surface as some of the positive sites of the polymer are balancing the negative charge of clay and other positive sites are neutralised by ${Cl}^{-}$ ions; the latter sites are randomly distributed in the interlayers. Chloride anions are exchanged with other anions, such as perchlorate, nitrate, and chromate, during anion adsorption. In addition, PDDA polymer is coiled in the interlayers (Figure 1), and hence, there is no uniform distribution of the sites to get uniform uptake, unlike surfactant intercalated into clay, where the surfactant chains are confined rigidly in the interlayers. Thus, the anion exchange sites in organoclays are relatively uniform, and the anion exchange in organoclays follows multilayer adsorption. Among all the Langmuir models fitted with P-MT-W, P-MT-C and P-Mt-J, the P-Mt-J showed a better correlation coefficient than the other montmorillonite-PDDA nanocomposites. The reason might be that the PDDA cations were adsorbed not only in the interlayers but also on the outside surfaces because they had been better exfoliated during the synthesis process.

The Freundlich isotherm model fitted well with all the MT-PDDA nanocomposites. This model assumes heterogeneous adsorption due to the diversity of adsorption sites. Mt-PDDA nanocomposites, indeed, have heterogeneous adsorption sites, as described above. The *n* value indicates the degree of nonlinearity between solution concentration and adsorption. When the value of 1 × n^−1^ in the adsorption isotherm is less than unity, it implies a heterogeneous surface structure with minimal interaction between the adsorbed atoms.

### 3.5. Kinetics Studies

**Figure 6:**
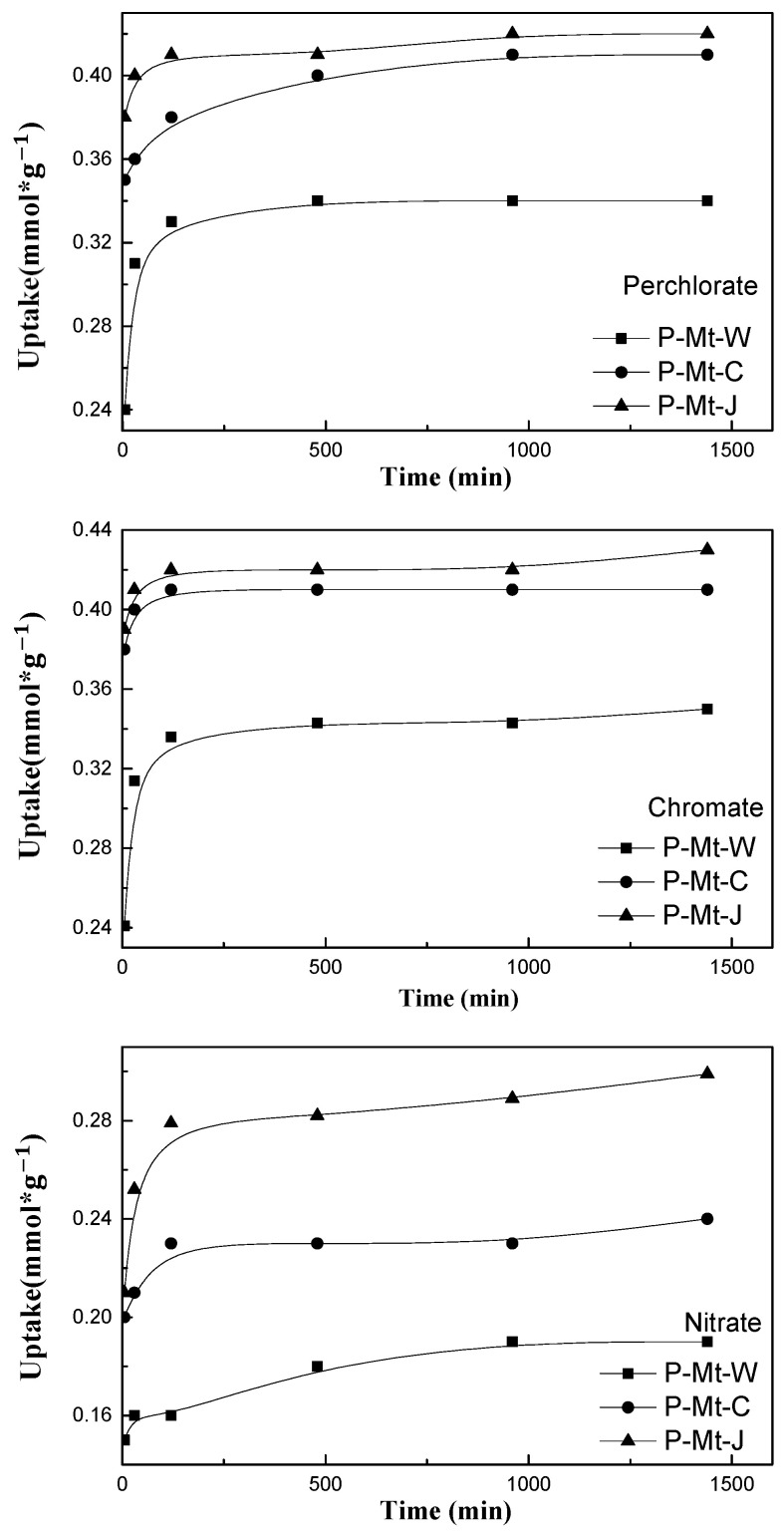
Kinetics of perchlorate, chromate and nitrate uptake by P-MT-W, P-MT-C and P-Mt-J nanocomposites.

The data on the adsorption kinetics of perchlorate, chromate, and nitrate onto Mt-PDDA nanocomposites are provided in Figure 6. The kinetic results showed that the uptake rates of perchlorate, chromate and nitrate were very high. The perchlorate, chromate, and nitrate uptakes by Mt-PDDA nanocomposites rose quickly in the first 30 min to 0.39 $mmol\cdot g^{-1}$, 0.38 $mmol\cdot g^{-1}$ and 0.36 $mmol\cdot g^{-1}$ by P-Mt-J respectively, but only a small increase occurred after 30 min until the end of the experiments. However, the adsorption of perchlorate, chromate and nitrate reached a plateau after 2 h when approximately 80%, 80%, and 60% of perchlorate, chromate, and nitrate were removed from the solution.

The Lagergren pseudo-first-order and Lagergren pseudo-second-order kinetic models were used to better understand the adsorption mechanisms and kinetics [41,45,52]. For the pseudo-first-order process, the Lagergren equation is expressed as [52]:

(6) $$\frac{{dq}_{t}}{d_{t}}=k_{1}(q_{e}-q_{t})$$

**Figure 7:**
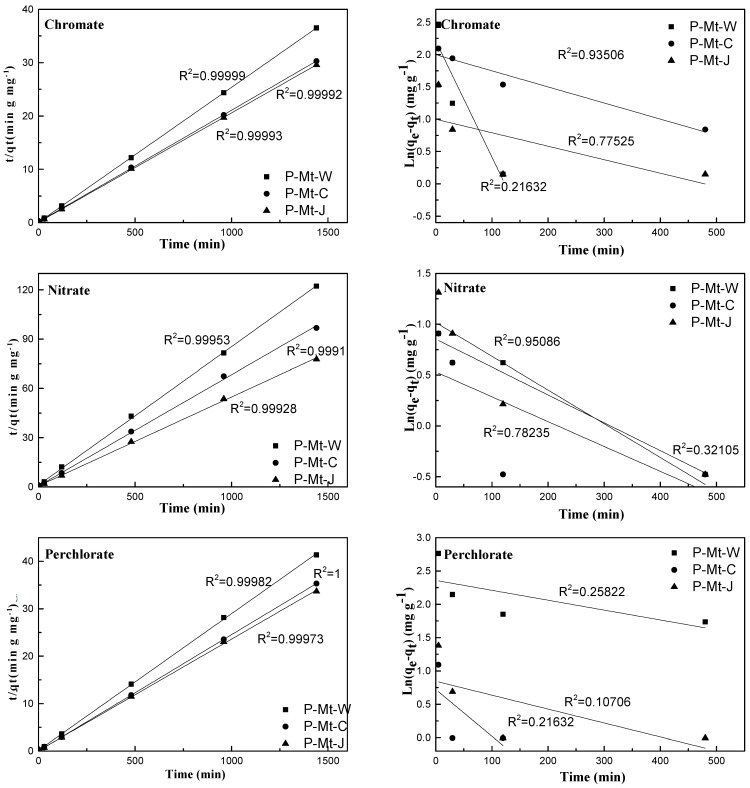
Pseudo-second order model (**left**) and Pseudo-first order model (**right**) for perchlorate, chromate, and nitrate by P-Mt-W, P-Mt-C and P-Mt-J nanocomposites.

Integrating this Equation with the conditions: $q_{t}=0$ at t=0 and $q_{t}=q_{t}$ at $q_{t}=t$ gives:

(7) $$\ln\left(q_{e}-q_{t}\right)=\ln q_{e}-k_{1}t$$

where $q_{t}$ $\left(mg\cdot g^{-1}\right)$ is the amount of adsorbed nitrate and perchlorate on the adsorbent at time t, $q_{e}$, the equilibrium sorption uptake, is extrapolated from the experimental data at time t = infinity and $k_{1}$ (min^−1^) is the rate constant of first-order adsorption. The pseudo-first-order model for perchlorate, chromate, and nitrate adsorption by Mt-PDDA nanocomposites is illustrated in Figure 7. The pseudo-second-order process can be written as follows [45]:

(8) $$\frac{t}{q_{t}}=\frac{1}{k_{2}{\left(q_{e}\right)}^{2}}+\frac{t}{q_{e}}$$

where $q_{e}$ $\left(mg\cdot g^{-1}\right)$ is the perchlorate, chromate, and nitrate concentrations at equilibrium and $q_{t}$ $\left(mg\cdot g^{-1}\right)$ is the perchlorate, chromate, and nitrate concentration at the time, t. Thus, by plotting $t\cdot {q_{t}}^{-1}$ versus t, the rate constant of second-order adsorption, $k_{2\;}\left(g\;{mg}^{-1}{min}^{-1}\right)$ and $q_{e}$ values can be determined. The pseudo-second-order model for the perchlorate, chromate and nitrate adsorption onto P-Mt-W, P-Mt-C and P-Mt-J is depicted in Figure 7. In addition, the kinetic parameters and rate constants for perchlorate, chromate and nitrate uptakes were calculated and reported in Table 3. The pseudo-second-order mechanism fitted well for perchlorate, chromate, and nitrate with correlation coefficients of 0.99, 0.99 and 0.99, respectively. The $q_{e}$ ($mg\;g^{-1}$) and $k_{2}\;\left(g\;{mg}^{-1}{min}^{-1}\right)$ values for nitrate were much higher in comparison to the experimental results for nitrate (Table 3). The greater equilibrium concentrations of nitrate are possible because the nitrate has a lower hydration energy than most other anions in natural water, such as chloride, bicarbonate and sulfate (Marcus, 1991 [53]), and hence they are not preferred by the hydrophilic Mt-PDDA polymer composites. The pseudo-second-order mechanism fitted well for perchlorate, chromate, and nitrate. This indicated that this sorption system is a pseudo-second-order reaction, implying the rate-limiting step may be chemical sorption involving valency forces through the sharing or exchange of electrons between sorbent and sorbate [54].

### 3.6. The Effects of Competitive Anions

**Figure 8:**
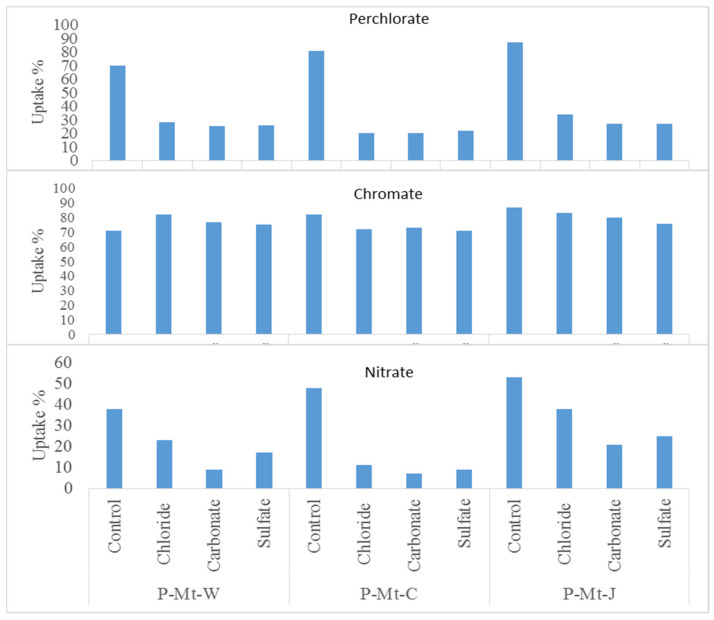
Effect of competitive anions on perchlorate, chromate, and nitrate uptakes by P-Mt-J, P-Mt-C and P-Mt-W nanocomposites.

The effects of major naturally occurring monovalent and divalent anions on the removal of nitrate, perchlorate, and chromate by different types of Mt-PDDA nanocomposites were determined and are shown in Figure 8. When the competing ions of chloride, carbonate and sulfate were present at ten times the concentration of either perchlorate chromate or nitrate, the uptake of chromate was practically unaffected, while perchlorate or nitrate uptake was significantly reduced when expressed either as percentages or Kd values with all the Mt-PDDA nanocomposites. All the nanocomposites, P-Mt-W, P-Mt-C, and P-Mt-J, showed the same trend of reduced uptake with both perchlorate and nitrate, i.e., severe competition by anions of chloride, carbonate, and sulfate when they were present at ten times the concentration of perchlorate and nitrate. However, the presence of chloride, sulfate and carbonate anions had a negligible effect on the chromate uptake capacity of P-Mt-W, P-Mt-C, and P-Mt-J nanocomposites, suggesting a high selectivity for the removal of chromate from aqueous solutions containing different anions. The inhibitory effect of selected anions on the removal efficiency of chromate, nitrate, and perchlorate by Mt-PDDA nanocomposites was about the same. These results are consistent with the Hofmeister series of anions based on the free energy of hydration. The Gibbs free energy values of hydration of the anions are shown in Table 4. The energy of hydration of each anion is as follows: ${ClO}_{4}^{-}<{NO}_{3}^{-}<{Cl}^{-}<{CrO}_{4}^{2-}<{SO}_{4}^{2-}<{CO}_{3}^{2-}$. The higher hydration energy of an anion indicates that it will take more energy to break the energy shell for dehydration.

**Figure 9:**
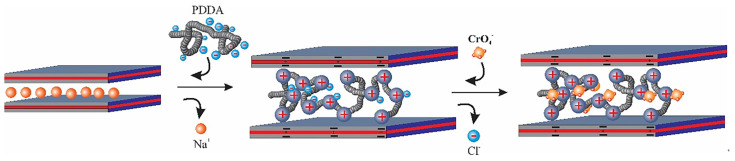
Schematic of chromate uptake by Mt-P DDA nanocomposite.

In the case of organoclay, the neutral surfactant with ${Cl}^{-}$ is entrapped by negatively charged clay layers and surfactant cations in the interlayers [55], which is schematically shown in Figure 9. Therefore, the ${Cl}^{-}$ anion is in a hydrophobic environment between organic cations. Thus, it is easy for organoclay to take up less hydrated ions, like perchlorate and nitrate, which can easily go into the interlayers because organoclay is a water-repelling type of material and hence, there is repulsion of hydrated ions. However, the Mt-PDDA- nanocomposites (Figure 1) are hydrophilic materials, and hence, there is not much preference for less hydrated ions like nitrate, chloride, and perchlorate when excess competing ions are present along with these ions. In the presence of mixed competing ions of sulfate, carbonate, or chloride, Mt-PDDA nanocomposites take up the hydrated ions of chromate preferentially. Therefore, Mt-PDDA nanocomposites have a higher selectivity for highly hydrated anions, like chromate, but not for less hydrated anions, such as perchlorate and nitrate (Figure 8).

## 4. Conclusions

The P-Mt-J Mt-PDDA nanocomposite showed the best perchlorate, chromate, and nitrate uptake with 0.40 mmol/g, 0.44 mmol/g and 0.299 mmol/g, respectively, because Mt from Japan has a higher total charge or higher CEC than the other two montmorillonites used in this study. With the higher charge of the Mt from Japan, more PDDA was intercalated into its interlayers. The higher the PDDA polymer content in the clay interlayers, the greater its anion exchange capacity. The uptakes of nitrate, perchlorate, and chromate by the Mt-PDDA nanocomposites could be well described using the Freundlich isotherm while their uptake kinetics fitted well to the pseudo-second-order model. The kinetics study indicated that nitrate, perchlorate, and chromate uptake kinetics were found to be fast as equilibrium was reached within 4 h in the Mt-PDDA nanocomposites. The Mt-PDDA nanocomposites exhibited selective uptake of chromate in the presence of 10 times excess equivalent concentration of competing anions such as ${Cl}^{-}$, ${SO}_{4}^{2-}$ and ${CO}_{3}^{2-}$. However, perchlorate and nitrate did not show very high selectivity when ten times excess equivalent concentration of competing anions such as ${Cl}^{-}$, ${SO}_{4}^{2-}$ and ${CO}_{3}^{2-}$ were present because these two anions are less hydrated than chromate. The presently developed Mt-PDDA nanocomposites may be useful for the remediation of hazardous anions from water and soil. These nanocomposite materials can also be potentially used for drinking water filtration.

## Figures and Tables

**Table 1:** The uptake of perchlorate, chromate and nitrate by P-Mt-W, P-Mt-C and P-Mt-J nanocomposites.

Sample	Solution	Removal (%)	Uptake (mmol/g)
P-Mt-W	1 mM Perchlorate	71	0.350 ± 0.020
P-Mt-C	1 mM Perchlorate	81	0.400 ± 0.010
P-Mt-J	1 mM Perchlorate	87	0.440 ± 0.001
P-Mt-W	1 mM Chromate	66	0.338 ± 0.009
P-Mt-C	1 mM Chromate	82	0.413 ± 0.017
P-Mt-J	1 mM Chromate	84	0.423 ± 0.005
P-Mt-W	1 mM Nitrate	38	0.189 ± 0.002
P-Mt-C	1 mM Nitrate	48	0.239 ± 0.004
P-Mt-J	1 mM Nitrate	53	0.299 ± 0.002

50 mg of material was added into 25 mL solutions and equilibrated for 24 h.

**Table 2:** Langmuir and Freundlich parameters for perchlorate, chromate and nitrate uptakes by P-Mt-W, P-Mt-C and P-Mt-J nanocomposites.

Anion	Material	Langmuir Model			Freundlich Model		
		$R^{2}$	$Q_{o}\left(mmol\;g^{-1}\right)$	$b\left(L\;mg^{-1}\right)$	$R^{2}$	K	1/n
Perchlorate	P-Mt-W	0.56	69.44	0.024	0.92	2.46	0.73
	P-Mt-C	0.16	117.6	0.015	0.82	1.87	0.89
	P-Mt-J	0.81	188.7	0.011	0.99	2.52	0.88
Chromate	P-Mt-W	0.84	59.17	0.018	0.93	2.01	0.67
	P-Mt-C	0.81	161.3	0.017	0.94	4.69	0.72
	P-Mt-J	0.82	175.4	0.047	0.98	11.0	0.65
Nitrate	P-Mt-W	0.95	17.18	0.048	0.99	1.82	0.49
	P-Mt-C	0.97	25.32	0.043	0.99	1.96	0.58
	P-Mt-J	0.99	30.67	0.064	0.99	2.94	0.59

**Table 3:** Kinetic parameters for perchlorate, chromate and nitrate uptakes by P-Mt-W, P-Mt-C and P-Mt-J—nanocomposites.

Material	Anion	Pseudo-Second Order Model			Pseudo-First Order Model	
		$R^{2}$	$k_{2}(g\;{mg}^{-1}\;{min}^{-1})$	$q_{e}(mg\;{mg}^{-1})$	$R^{2}$	$k_{1}\left(g\;{mg}^{-1}\;{min}^{-1}\right)$
P-Mt-W	Nitrate	0.99	0.0250	39.78	0.26	0.0031
	Perchlorate	0.99	0.0254	40.77	0.22	0.0179
	Chromate	0.99	0.0855	42.76	0.96	0.0027
P-Mt-C	Nitrate	0.99	0.0251	76.84	0.21	0.007
	Perchlorate	0.99	0.0212	92.66	0.94	0.0025
	Chromate	0.99	0.0666	94.92	0.32	0.0024
P-Mt-J	Nitrate	0.99	0.233	11.78	0.10	0.0109
	Perchlorate	0.99	0.0206	14.88	0.77	0.0021
	Chromate	0.99	0.0383	26.04	0.78	0.0033

**Table 4:** The radius, r, width of hydration shell, ∆r, number of water molecules in this shell, n and experimental values, ${∆}_{hyd}G^{*}$, of the molar Gibbs energies of hydration of ion [53].

Ion	r/nm	∆r/nm	n	${∆}_{hyd}G^{*}/kJ\;{mol}^{-1}$
${ClO}_{4}^{-}$	0.200	0.033	1.8	−280
${NO}_{3}^{-}$	0.179	0.044	2.0	−300
${Cl}^{-}$	0.181	0.043	2.0	−340
${CrO}_{4}^{2-}$	0.240	0.039	3.0	−950
${SO}_{4}^{2-}$	0.230	0.043	3.1	−1080
${CO}_{3}^{2-}$	0.178	0.076	4.0	−1315

## Data Availability

Data are contained within the article.
